# Amelanotic subungual melanoma mimicking a glomus tumor: A diagnostic pitfall requiring thumb amputation

**DOI:** 10.1016/j.jdcr.2026.04.058

**Published:** 2026-05-05

**Authors:** Kianna Thelen, Alexandra Job, Emily Storm, Ali Jassim

**Affiliations:** a University of South Dakota Sanford School of Medicine, Sioux Falls, South Dakota; b Department of Pathology, Sanford USD Medical Center, Sioux Falls, South Dakota

**Keywords:** amelanotic melanoma, diagnostic pitfall, nail unit tumor, PRAME, SOX10, subungual melanoma, thumb amputation

## Introduction

Subungual melanoma is a rare malignancy, accounting for approximately 0.7% to 3.5% of all melanomas.^1^^,^^2^ Among subungual melanomas, amelanotic cases are uncommon, with prior reports estimating an incidence ranging from approximately 15% to 33%.^3^ Nail unit melanoma most often presents in older adults, typically in patients over 50 years of age.^4^ Amelanotic variants present an even greater diagnostic challenge due to their lack of pigmentation and frequent absence of staining with conventional melanocytic markers. In clinical settings where prior nail trauma or occupational exposure may cloud the presentation, these tumors are particularly prone to misdiagnosis. We describe a case of amelanotic subungual melanoma initially presumed to be a glomus tumor, ultimately necessitating thumb amputation for local control.

## Case report

**Fig 1:**
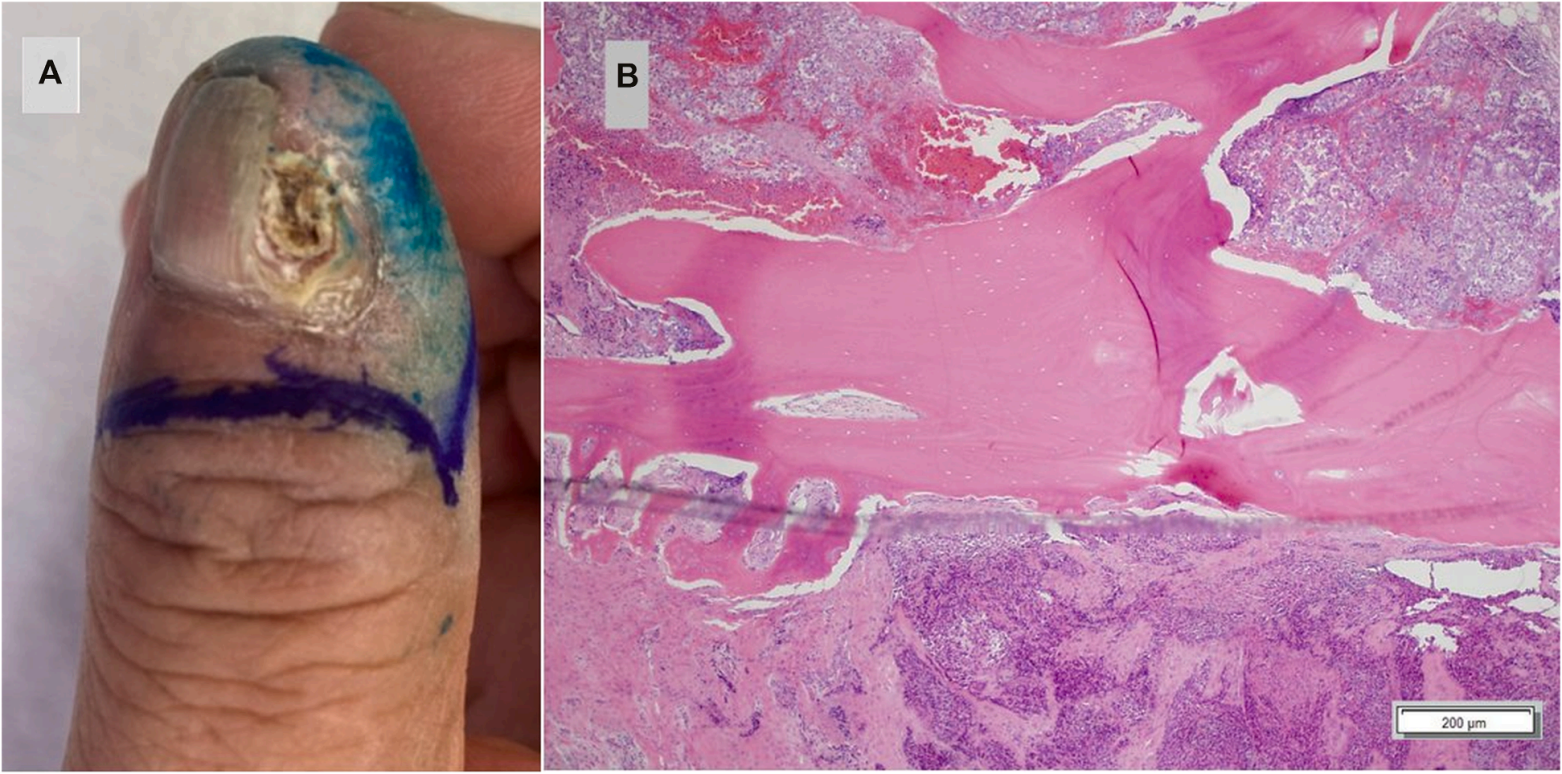
Definitive surgery reveals extensive disease. **A,** Preoperative appearance demonstrates nail-bed deformation without pigment. **B,** Representative H&E sections from the amputated digit show deep invasion extending into subcutaneous tissue with osseous involvement (×4).

A 62-year-old Hispanic male with a medical history of prediabetes initially presented to an orthopedic surgeon on September 13, 2023, for evaluation of persistent pain and swelling of the left thumbnail. He is currently employed as a construction worker and initially attributed his symptoms to occupational trauma after striking his thumb with a hammer. He had previously been treated for presumed paronychia with drainage and partial nail plate removal; however, he continued to notice a firm subungual mass that progressively enlarged over 6 months, causing significant pain and interfering with daily activities. On examination, there was partial obstruction of the radial aspect of the nail plate by a flesh-colored mass at the nail bed, without ulceration or drainage (Fig 1, *A*). Sensation was intact and active thumb interphalangeal joint motion was preserved. Plain radiographs demonstrated questionable bony involvement, and a magnetic resonance imaging performed on October 11, 2023, favored the diagnosis of a glomus tumor. This radiographic impression can occur when a well-circumscribed subungual lesion demonstrates T2 hyperintensity with avid postcontrast enhancement, features that are typical of vascular glomus tumors and may therefore contribute to diagnostic anchoring based on location and radiographic appearance rather than histology.^5^

Given functional impairment, persistent pain, and imaging suggestive of a benign glomus tumor, the patient underwent excision of the left thumb mass with partial matrixectomy and local soft tissue rearrangement under wide-awake local anesthesia on October 23, 2023, as a therapeutic procedure that also served as an excisional biopsy. Intraoperative findings included a flesh-colored, slightly bluish mass involving approximately 50% of the nail bed with obliteration of the overlying nail plate but no gross evidence of bone invasion. The mass and underlying abnormal tissue were sharply excised down to the level of the distal phalanx, which appeared intact.

**Fig 2:**
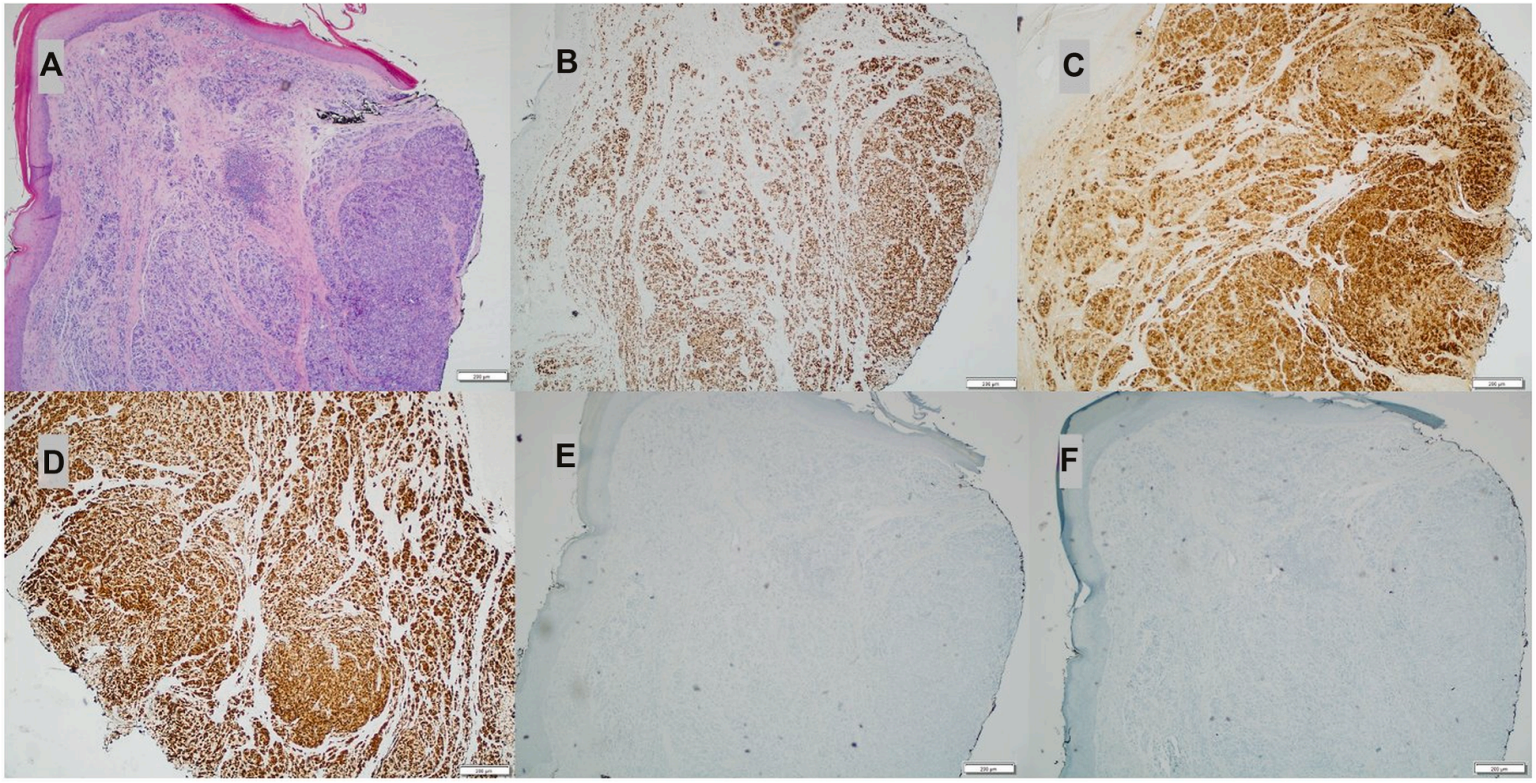
Immunohistochemical stains identify melanoma: On initial excision, the tumor was noted to be poorly differentiated and amelanotic on H&E stain **(A)**. Among the immunohistochemical stains used to characterize the lesion, the malignant cells were strongly positive for melanoma markers PRAME **(B),** S100 **(C),** and SOX10 **(D)**. Surprisingly, the tumor was negative for HMB45 **(E)** and MART-1 **(F),** which are classically positive in melanoma. All images were acquired at ×4 magnification.

**Fig 3:**
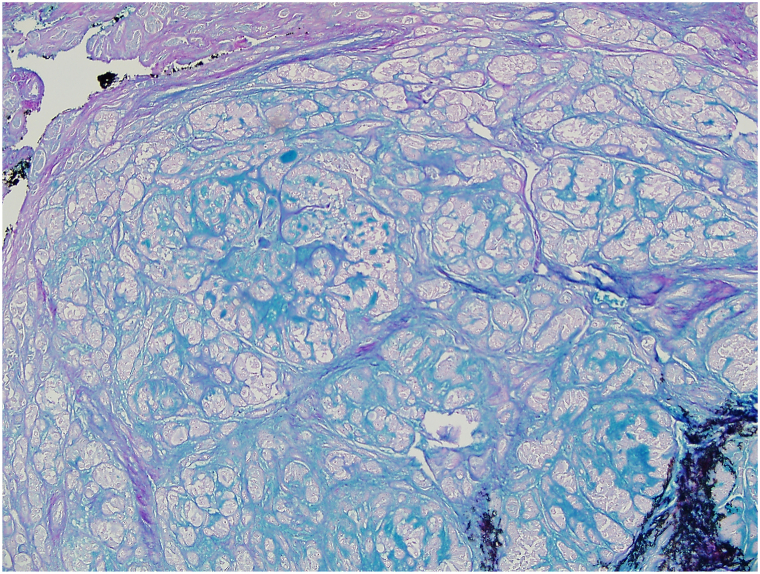
Colloidal iron stain (×10) highlights intracytoplasmic mucin-like material within tumor cells.

Histopathologic evaluation of the excised tissue revealed malignant amelanotic melanoma with epithelioid features and a poorly differentiated, predominantly nested growth pattern; an in situ/junctional component was not identified. The Breslow depth was at least 3.5 mm, as the tumor extended to the base of the specimen, with involvement of deep and peripheral margins; the mitotic rate was 2/mm^2^ and ulceration was not identified. No regression, lymphovascular invasion, or neurotropism was seen. Immunohistochemical staining was positive for PRAME, S100, and SOX10 but negative for MART-1 and HMB45 (unusual for melanoma), as well as ERG and pan-keratin (Fig 2). Colloidal iron staining highlighted intracytoplasmic mucin-like material (Fig 3), a rare finding in melanoma; this may represent true acidic mucopolysaccharide deposition versus artifactual/degenerative change, and it did not preclude diagnosis in this case given supportive morphology and immunophenotype. PD-L1 immunostaining showed 0% expression in the tumor and immune cells (combined positive score 0.0), and molecular testing for BRAF V600E mutation was negative.

Staging workup, including positron emission tomography-computed tomography, demonstrated no evidence of nodal or distant metastasis. To achieve local control and obtain negative margins in the setting of deep nail-unit melanoma with concern for distal phalanx involvement, the patient underwent left thumb disarticulation at the interphalangeal joint with sentinel and non-sentinel left axillary lymph node biopsy on December 5, 2023.

Final pathology from the amputation specimen confirmed residual malignant melanoma extending deep into the dermis, subcutaneous fat, and bone with a measured Breslow depth of approximately 7.2 mm (Fig 1, *B*). Resection margins were negative for residual tumor, and no definite vascular or lymphatic invasion was seen. Sentinel and nonsentinel left axillary lymph nodes were benign, with MART-1, HMB45, and PRAME immunostains negative for metastatic melanoma.

Based on these findings, the tumor was staged as Stage IIB (pT4a pN0 cM0) melanoma of the left thumb. The patient’s case was discussed in a multidisciplinary setting, and options, including observation versus adjuvant systemic therapy, were reviewed according to the National Comprehensive Cancer Network guidelines. The patient elected to proceed with adjuvant immunotherapy rather than enrollment in a clinical trial. He began treatment with nivolumab at 240 mg every 2 weeks starting January 11, 2024, with plans to monitor tolerance and titrate to 480 mg every 4 weeks if appropriate. He tolerated the initial dose well and continues to follow up for ongoing surveillance and management.

## Discussion

Subungual amelanotic melanoma is rare and easily misdiagnosed due to its nonpigmented appearance and tendency to mimic benign lesions such as glomus tumors, inclusion cysts, or pyogenic granulomas.^6^ Published case reports underscore that amelanotic subungual melanoma is frequently misdiagnosed clinically and treated for alternative inflammatory or infectious nail conditions, contributing to delayed definitive management. Prior reports describe clinical misdiagnoses including onychomycosis, lichenoid nail disease (lichen planus–like), chilblains/pernio-related changes, and nonspecific onychodystrophy, which can delay biopsy and definitive treatment.^7^, ^8^, ^9^, ^10^ In this case, misleading clinical and radiographic features contributed to an initial misdiagnosis and delay in definitive diagnosis.

Histopathologic confirmation can be difficult because conventional melanocytic markers such as MART-1 and HMB45 are often negative.^11^^,^^12^ PRAME has emerged as a helpful marker with high sensitivity and specificity for melanocytic malignancies, especially in diagnostically challenging or amelanotic presentations.^12^ SOX10 remains an essential adjunct marker when conventional markers are negative or equivocal.^12^^,^^13^ In this patient, PRAME and SOX10 positivity were crucial to reaching the correct diagnosis (Table I).^11^^,^^12^

**Table I:** Common immunohistochemical stains used in amelanotic melanoma and diagnostic considerations^11^, ^12^, ^13^

Stain/M arker	Typical role in amelanotic melanoma	This case	Common pitfall
S100	Highly sensitive melanocytic marker; useful screening stain	Positive (diffuse strong)	Less specific; can label non-melanocytic tumors
SOX10	Highly sensitive nuclear melanocytic marker; helpful when MART-1/HMB45 are negative	Positive (diffuse nuclear)	Can label some neural crest–derived tumors
PRAME	Supports melanoma diagnosis; helpful adjunct in challenging/amelanotic cases	Positive (diffuse nuclear)	Not universally positive; interpret with morphology and other markers
MART-1 (Melan-A)	Traditional melanocytic marker	Negative	May be negative in amelanotic melanoma
HMB45	Traditional melanocytic marker	Negative	May be negative in amelanotic melanoma
Pan-keratin	Helps exclude epithelial neoplasms	Negative	—
ERG	Helps exclude vascular neoplasms	Negative	—

Amelanotic melanomas may show reduced expression of MART-1 and HMB45, increasing diagnostic reliance on an expanded panel including S100, SOX10, and PRAME.^12^ Typical immunoprofile: S100 and SOX10 often remain positive; HMB45 and MART-1 can be reduced or negative; PRAME may support diagnosis when positive.

The colloidal iron–positive intracytoplasmic mucin-like material is an uncommon finding in melanoma and can raise consideration of myxoid/chondroid change or artifact; in our case, the diagnosis was supported by the overall morphology and PRAME/S100/SOX10 immunophenotype despite absent MART-1/HMB45 staining.^12^

Even when an early biopsy is performed, nail-unit melanomas can present a histologic diagnostic pitfall: some cases show poorly differentiated or spindle-cell morphology, desmoplastic features, or limited junctional disease, which may obscure melanocytic differentiation on routine sections and increase reliance on a broad immunohistochemical panel.^12^

Intracytoplasmic mucin-like material is uncommon in melanoma; when present, it may be seen in melanomas with myxoid change or signet-ring–like cytoplasmic vacuolization and can bias interpretation toward mucin-producing carcinoma.^14^^,^^15^ In our case, negative cytokeratin staining with strong melanocytic marker expression supported melanoma despite this unusual feature.

The tumor’s lack of PD-L1 expression and absence of a BRAF V600E mutation limited systemic therapeutic options, reinforcing the importance of achieving negative margins through appropriate surgery. Despite negative PD-L1 expression, adjuvant PD-1 blockade (nivolumab) was selected for this resected Stage IIB melanoma given evidence of recurrence-risk reduction with PD-1 inhibitors in high-risk stage II disease, and because PD-L1 status is not required to pursue adjuvant anti–PD-1 therapy.^16^ When persistent or painful nail unit lesions do not resolve with conservative measures and imaging suggests a benign process, early biopsy remains critical to avoid progression requiring radical interventions such as amputation.

The increase in measured Breslow depth from the initial specimen (≥3.5 mm, tumor to base) to the amputation specimen (7.2 mm with osseous invasion) likely reflects undersampling inherent to partial nail-unit excisions and matrixectomy specimens, in which the deepest extent of nail-unit melanoma may not be fully captured. This limitation reinforces the importance of adequate sampling and margin assessment when melanoma is in the differential.^12^^,^^17^

This case highlights a clinical diagnostic pitfall in the nail unit; overattributing to trauma and a benign working diagnosis, while also underscoring that nail-unit melanoma may present histologic diagnostic challenges that warrant expanded immunohistochemistry.^12^

## Conclusion

Amelanotic subungual melanoma, although rare, must remain an important diagnostic consideration in nail unit pathology. Clinicians should maintain a high index of suspicion for melanoma in persistent or painful subungual lesions, even when imaging supports a benign etiology. Early biopsy and comprehensive immunohistochemical staining are essential to ensure timely diagnosis and appropriate management, which may prevent the need for extensive surgical procedures such as amputation.

## Conflicts of interest

None disclosed.
